# Automatic Semicircular Canal Segmentation of CT Volumes Using Improved 3D U-Net with Attention Mechanism

**DOI:** 10.1155/2021/9654059

**Published:** 2021-09-07

**Authors:** Hongcheng Wu, Juanxiu Liu, Gui Chen, Weixing Liu, Ruqian Hao, Lin Liu, Guangming Ni, Yong Liu, Xiaowen Zhang, Jing Zhang

**Affiliations:** ^1^MOEMIL Laboratory, School of Optoelectronic Science and Engineering, University of Electronic Science and Technology of China, No. 2006, Xiyuan Avenue, West Hi-Tech Zone, Chengdu 611731, China; ^2^State Key Laboratory of Respiratory Disease, Department of Otolaryngology, Head & Neck Surgery, Laboratory of ENT-HNS Disease, First Affiliated Hospital of Guangzhou Medical University, Guangzhou, China

## Abstract

The vestibular system is the sensory apparatus that helps the body maintain its postural equilibrium, and semicircular canal is an important organ of the vestibular system. The semicircular canals are three membranous tubes, each forming approximately two-thirds of a circle with a diameter of approximately 6.5 mm, and segmenting them accurately is of great benefit for auxiliary diagnosis, surgery, and treatment of vestibular disease. However, the semicircular canal has small volume, which accounts for less than 1% of the overall computed tomography image. Doctors have to annotate the image in a slice-by-slice manner, which is time-consuming and labor-intensive. To solve this problem, we propose a novel 3D convolutional neural network based on 3D U-Net to automatically segment the semicircular canal. We added the spatial attention mechanism of 3D spatial squeeze and excitation modules, as well as channel attention mechanism of 3D global attention upsample modules to improve the network performance. Our network achieved an average dice coefficient of 92.5% on the test dataset, which shows competitive performance in semicircular canals segmentation task.

## 1. Introduction

**Figure 1:**
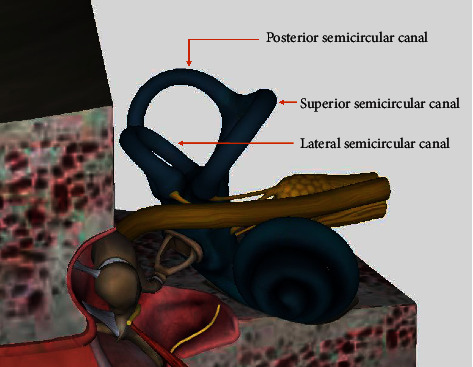
Structure of the semicircular canal in the inner ear.

The lifetime prevalence of vertigo among adults is 7.4%, with a one-year prevalence of 4.9% and a one-year incidence of 1.4% [1]. Dysfunction of the vestibular system is one of the most essential causes for vertigo. The vestibular apparatus is small, beautifully formed, and located on the deep side of the temporal bone that controls the human sense of balance and movement [2]. Humans can perceive head rotation, angular acceleration, and orientation in space due to the specific structures of the vestibular system: the otolith organs and semicircular canals [3]. Semicircular canal encodes head rotational velocity and provides input to the vestibule-ocular reflex, vestibulocollic reflex, vestibulospinal system, vestibuloreticular system, cerebellum, and cortex [4]. Benign paroxysmal positional vertigo (BPPV) is the most common cause of vertigo due to the vestibular system disorders. The mechanism of BPPV has been attributed to cupulolithiasis or canalithiasis, which can affect the function of semicircular canal and thus cause vertigo [5]. As shown in Figure 1, the semicircular canal consists of a superior semicircular canal, a posterior semicircular canal, and a lateral semicircular canal. Among medical imaging technologies, Computed Tomography (CT) technology is an important method for inner ear disease diagnosis. However, semicircular canals have sophisticated structure and small volume, which accounts for less than 1% of total area in a single CT image [6], and it brings huge difficulties to diagnosis. Automatic segmentation of the semicircular canals in CT images can help screen out diseases such as malformation and superior semicircular canal bony dehiscence. In addition, the angle of the segmented semicircular canal can be used to customize the reduction therapy during the treatment of otolithiasis. The anatomical semicircular canal model is of great significance for studying the mechanism of the vestibule. Thus, it is meaningful to automatically segment the semicircular canals, which provide precious anatomical training resources for doctors.

Recently, a variety of advanced algorithms have been proposed for medical image processing, ranging from traditional method to machine learning and deep learning [7]. Some traditional methods, like the threshold method, require manually setting one or several suitable thresholds. However, with limited spatial resolution or motion artifacts in CT imaging, some voxels in CT images contain a mixture of many tissue types instead of just a single tissue type [8]. Thus, multiple thresholds setting is required, which is time-consuming and has poor generalization performance. The region growing method is another traditional method that requires selecting suitable seed points, and the segmentation results are highly correlated with them. However, it is challenging to choose appropriate seed points manually. The patterns of regional growth are also sensitive to noise, which may cause the extracted region to blank or link the separate region under the case of local effect [9]. Clustering is a machine-learning-based technique for image segmentation. The principle of clustering is simple and its convergence speed is fast, which is one of the most important reasons why the industry adopts it a lot. Nevertheless, the clustering method is sensitive to the local intensity changes [10]; its performance is unavoidably interfered by noise in small organ segmenting tasks of CT volumes.

Although the methods mentioned above are relatively simple to implement, using these manual segmentation methods to segment small organs in the CT volumes is time-consuming and not task-specific [11], which requires a sophisticated knowledge base of anatomy. In addition, small organ segmentation tasks require high precision, and in that case, these traditional methods are not suitable. In recent years, with the fast development of artificial intelligence technology represented by deep learning, additional technical methods for the precise segmentation of medical images have been proposed [6]. Numerous deep learning network structures are proposed in different organ segmentation tasks, such as skin [12], brain tumor [13], heart [10], lung [14], and pancreas [15, 16] segmentation.

Medical image segmentation based on deep learning technology can be roughly divided into two categories, namely, 2D CNN- (Convolutional Neural Network) based methods and 3D CNN-based methods. Methods are based on 2D CNN segment volumetric CT or Magnetic Resonance Imaging (MRI) data in a layer-by-layer manner. For example, Fully Convolutional Network (FCN) [17] substitutes fully connected layers with convolutional layers, which increases the generalization ability of the network and improves training efficiency at the same time. However, the FCN architecture tends to omit the detailed information of the image due to pooling layers. Thus, some researches have been investigated to improve the accuracy of segmentation. Later on, a U-Net [18] architecture was proposed. This architecture improves the segmentation accuracy and addresses the problem of gradient vanishing, becoming one of the most popular architectures in segmentation tasks of medical images [6].

Volumetric data accounts for a large portion of medical image modalities, such as 3D computed tomography (CT), 3D Magnetic Resonance images, and 3D ultrasound [19]. Although 2D CNNs have achieved a great breakthrough in slice-based medical image segmentation tasks, the slicing strategy in 2D CNNs segmentation pipelines hardly takes full advantage of the 3D spatial information existing in the volumetric CT images to achieve competitive segmentation results as 3D CNNs. To utilize the entire 3D medical image, several 3D segmentation networks have been proposed, including 3D U-Net [20], V-Net [21], Residual Symmetric U-Net [22], DenseVoxNet [23], and VoxResNet [19]. These 3D architectures can make full use of the 3D contexture information and greatly improve their capability to segment objects from volumetric data.

Among the networks mentioned, 3D U-Net is most widely used. 3D U-Net adopts a bilateral symmetric architecture including an encoder path to capture context information and a symmetric decoder path to recover spatial position, and in this way, it provides a full-resolution segmentation. The encoder and decoder are connected by skip connections [6]. However, the skip connections used in 3D U-Net may cause the loss of detailed information due to the gap between low-level features and high-level features [24]. To overcome the shortage of 3D U-Net, we introduce attention mechanism to the 3D U-Net, including 3D spatial squeeze and excitation (3D spatial SE) [25] module, as well as 3D Global Attention Upsample (3D GAU) [26] module. The 3D spatial SE module is a kind of spatial attention module that captures the spatial dependencies between any two positions of the feature maps from the encoder, aiming at guiding the network to “look where.” The 3D GAU module is deployed at the decoder path, which is a kind of channel attention module. This module uses low-level information to help high-level features recover images detail, which avoids the shortcomings of original skip connections used in 3D U-Net. By adding the attention mechanism, our network is capable of increasing receptive field and segmenting small objects more effectively. Experimental results show that the proposed network achieves the highest accuracy in the semicircular canal segmentation task.

For the clinical field, surgeons routinely review the CT volumes as multiplanar two-dimensional representations, although the CT datasets are inherently volumetric. Traditional methods based on handcraft features, as well as most of the previous deep learning methods, still process two-dimensional images layer by layer. It is difficult for these methods to meet clinical requirements in terms of speed and accuracy. Our 3D deep learning network with attention mechanism can segment small organs like semicircular canals accurately, quickly, and fully automatically using three-dimensional data, which is a further step to computer-aided otopathy CT image processing. Specifically, the segmentation results can be used to establish a virtual reality surgical simulation of the inner ear surgery or help to customize the reduction therapy during the treatment of otolithiasis.

We make the following contributions:

1. We propose a novel network and apply it to the semicircular canal CT segmentation task. This method can automatically separate the semicircular canals from multiple volumes, assisting radiologists in clinical diagnosis.
2. We adapt attention mechanism to the 3D U-Net architecture. The 3D spatial SE modules of spatial attention mechanism are deployed at the encoder and the 3D GAU modules of channel attention mechanism are added at the decoder. The attention mechanism guides the network training process and improves model sensitivity to foreground pixels in the whole CT volumes without requiring complicated heuristics. Our proposed network solves the problem of information loss in skip connections of 3D U-Net, achieving great effects in segmenting small organs like the semicircular canal in the CT volumes.
3. Sufficient ablation experiments have been done to verify the effectiveness of the improvements based on 3D U-Net. We also compare the performance of our proposed network and the other state-of-the-art CNN architectures.
4. The semicircular canal can be segmented effectively with the proposed method, so can the other organs in the future, for example, facial nerve [27], cochleae [28], and spinal cord [29].

The rest of this article is organized as follows: Section 2 briefly reviews related works, and Section 3 details our network architecture, training method, and inference process in further depth. The dataset we used and the experiment results are introduced in Section 4. Finally, Section 5 and Section 6 present the discussion and conclusion, respectively.

## 2. Related Works

### 2.1. Traditional Segmentation Methods

Before the rapid development of deep learning, traditional methods like threshold method and region growing method are most widely used. Threshold method requires manually or automatically setting thresholds to segment targets from the background. It is suitable for segmenting high-contrast objects with sharp edges. However, it tends to be sensitive to noise and relies on image quality [30]. It is time-consuming and nearly impossible to manually find suitable thresholds in the whole 3D medical dataset [31]. Region growing method is very sensitive to the selection of seed points and noise. If the seed points are not selected properly, segmentation is prone to mistake background noise as the target. Such methods often require high contrast between the target area and the background [8]. In that way, due to the low contrast of CT images, threshold method and region growing method are not suitable for our scenario.

### 2.2. Machine-Learning-Based Methods

Clustering is a machine-learning-based technique for grouping similar data according to certain similarity criteria. K-means clustering and fuzzy C-means clustering (FCM) algorithms are two basic clustering segmentation algorithms in image processing [30]. Image segmentation methods based on fuzzy clustering and their improved algorithms have been widely used. Abdel-Maksoud et al. [32] proposed an image segmentation approach for accurate brain tumor detection using K-means clustering technique integrated with fuzzy C-means algorithm, improving the segmentation quality and accuracy in minimal execution time. However, the clustering method is too sensitive to noise and does not meet clinical requirements.

### 2.3. Deep-Learning-Based Segmentation Methods

2D CNN-based methods greatly improve segmentation accuracy compared with traditional segmentation methods. For example, Havaei et al. [33] proposed a two-way shallow network with different cascade structures for the segmentation of tumors in brain MRI images. This network achieved a dice score of 83.2% on the test dataset. Ronneberger et al. [18] proposed a U-Net architecture for cell segmentation. This architecture improves the segmentation accuracy and addresses the problem of gradient vanishing, so it becomes one of the popular architectures in segmentation tasks of medical images. By introducing new modules or improvements, many U-Net variants have been proposed. For example, Li et al. [34] modified the U-Net structure by deleting the crop operation and changing the loss function, which increases the processing speed of the network by 70% and enhances the performance of ore image segmentation. Weighted Res-UNet [35] is inspired by residual connections [36]. The residual connections are added in the encoding stage of U-Net. Weighted Res-UNet surpasses the baseline U-Net model on both the accuracy and sensitivity performance in the retinal vessel segmentation problem. To reduce the burden of deep network training, Tao et al. [37] combined the U-Net with residual network and changed the serial connection mode of convolution layer into a form of residual mapping and achieved better Mean Intersection over Union (MIoU) (0.928) in the precipitation cloud segmentation task. Zhou et al. [24] proposed U-Net ++ which redesigns the jump path to narrow the semantic gap between the feature maps of the encoding subnetwork and the decoding subnetwork, achieving an average IoU gain of 3.9 points over U-Net in liver and polyp CT image segmentation tasks.

However, there is massive missing spatial information in 2D slice-based medical data. To make full use of 3D medical images, several 3D segmentation networks have been proposed. 3D U-Net [20], an extension of 2D U-Net architecture [18], takes 3D volumes as input and processes them with corresponding 3D operations. This network can learn from sparsely annotated volumetric images and achieves good results for Xenopus kidney segmentation. To solve the problem of semicircular canal and other small organ segmentation, Li et al. proposed 3D-DSD [6], redesigned a 3D dense connection block and 3D multipool feature fusion scheme in the encoding stage, and adopted a 3D depth supervision mechanism in the decoding stage. The network improves the accuracy in temporal bone segmentation over 3D U-Net, achieving an average dice coefficient of 77.8% on their dataset. V-Net [21] uses a codec scheme and proposes a loss layer based on dice coefficients. The network helps to deal with situations where there is a strong imbalance between the number of foreground voxels and background voxels, and the author demonstrated the efficiency in the prostate segmentation task. Based on 3D U-Net, Residual Symmetric U-Net [22] is designed for 3D reconstruction of neurons from electron microscopic brain images. The network replaces concatenation joining by summation joining where the skip connections join the expanding path and avoids the border effect that may hurt accuracy. Huang et al. [38] introduced DenseNet for object recognition, which introduces direct connections from any layer to all subsequent layers to ensure the maximum information flow among layers, reducing the information loss. Song et al. [39] extended DenseNet and proposed Deep 3D-Multiscale DenseNet, which has a great effect on suppressing the overfitting problem of network training when the dataset is small. Yu et al. [23] extended DenseNet to cardiovascular three-dimensional segmentation and proposed DenseVoxNet. Their network achieved the best dice score of 0.931 ± 0.011. Chen et al. [19] proposed VoxResNet for key brain tissues segmentation in 3D MR images. To effectively train the deep network with limited training data, they seamlessly integrate multimodality and multilevel contextual information into the network, which helps harness the complementary information of different modalities and exploit the features of different scales.

## 3. Methods

In this section, we introduce the proposed 3D end-to-end architecture with 3D spatial SE modules and 3D GAU modules for semicircular canal segmentation.

### 3.1. The Proposed Network Architecture

Our proposed network is based on the 3D U-Net architecture which is widely used in medical image analysis. The 3D U-Net architecture is composed of an encoder and a decoder, which extracts low-level features and high-level features, respectively. The encoder extracts the features by a series of convolution and max-pooling layers, while the decoder recovers image resolution by deconvolution layers. However, the levels of features in the encoder path are much lower than those in the decoder path, so it is not conducive enough to make full use of the multiscale and multilevel features by simply using the skip connections to concatenate the feature maps from different paths. Therefore, we, respectively, introduce two attention modules to emphasize meaningful features along spatial and channel axes. 3D Spatial SE [25] module and 3D GAU [26] module are employed as the spatial attention module and the channel attention module, respectively, and they provide guidance for CNN to focus on the targets rather than the background efficiently.

**Figure 2:**
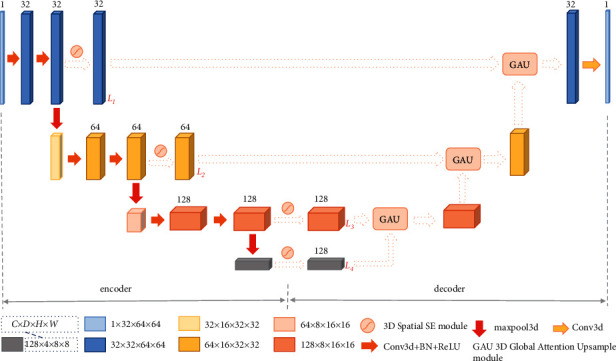
The architecture of the proposed network. The network structure is divided into two parts: encoder and decoder. Between the encoder and the decoder, we use the 3D spatial squeeze and excitation modules and 3D global attention upsample modules to replace the original skip connections used in 3D U-Net. The number on each feature block represents the channel, the size of the feature blocks in different colors are marked at the bottom, in *C* × *D* × *H* × *W* format, and *C*, *D*, *H*, and *W* represent the channel, depth, height, and width, respectively.

The network is shown in Figure 2. Encoder path consists of four layers, the first three of which contain two convolution operations (kernel: [3, 3, 3], stride: [1, 1, 1], channels: 32, 64, 128) each followed by a batch normalization and a rectified linear unit (ReLU). The max-pooling operations (kernel: [2, 2, 2], stride: [2, 2, 2]) are employed between each layer. We introduce 3D spatial SE modules at the end of each layer to spatially calibrate the features and get the four feature maps of *L*_1_, *L*_2_, *L*_3_, and *L*_4_ in Figure 2. In the decoder stage, low-level and high-level feature maps from adjacent layers are fused by several 3D GAU modules, and the cascaded 3D GAU modules restore the image size at the same time.

### 3.2. 3D Spatial SE Module

**Figure 3:**
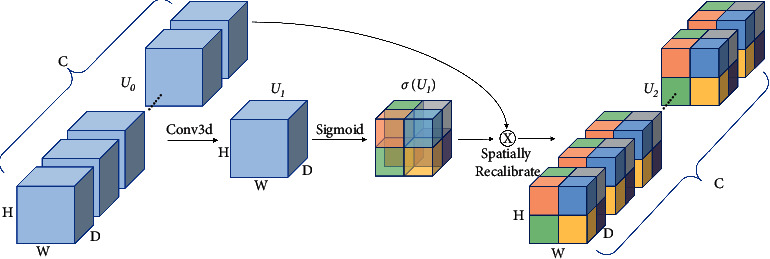
3D spatial SE module.

Squeeze and excitation (SE) attention block [40] is introduced to improve the accuracy of segmentation, which only excites channelwise. The Spatial SE attention block was designed based on the SE attention block, which “squeezes” along the channels and “excites” spatially [25]. Considering that the pixelwise spatial information of low-level features is more informative, we extend the Spatial SE module to 3D and introduce it into the encoder. The 3D spatial SE block is depicted in Figure 3. We consider the input feature map as follows:

(1) $$\begin{array}{c} U_{0}=\left[u^{1,1,1,1},u^{1,1,1,2},\ldots ,u^{h,i,j,k},\ldots ,u^{C,D,H,W}\right], \end{array}$$

where *u*^*h*,*i*,*j*,*k*^ ∈ *ℝ*, *h* ∈ {1,2,…, *C*}, *i* ∈ {1,2,…, *D*}, *j* ∈ {1,2,…, *H*}, and *k* ∈ {1,2,…, *W*}. *h* represents the channel location. *i*, *j*, and *k* represent the spatial location in the image. By a 3D convolution (kernel: [3, 3, 3], stride: [1, 1, 1], channels: 1) conv3d with output channel of one, the spatial squeeze operation is achieved, generating a projection tensor *U*_1_ ∈ *ℝ*^1×*D*×*H*×*W*^. This projection tensor passes through a sigmoid operation *σ*(·) to rescale activations to [0, 1], which is used to excite *U*_0_ spatially by a spatial-wise multiply operation. The definition can be formulated as follows:

(2) $$\begin{array}{c} U_{2}=\sigma \left(U_{1}\right)\times U_{0}. \end{array}$$

The spatial attention module tells where to focus, and the feature at a specific position is updated by aggregating features at all positions. This recalibration helps the network concentrate more on relevant spatial information and ignore the irrelevant ones.

### 3.3. 3D Global Attention Upsample Module

Our channel attention module is inspired by Pyramid Attention Network [26], in which 2D GAU module is used to merge the two feature maps that belong to adjacent layers and establish a connection between the high-level and low-level features. To reduce the category information lost in the low-level features and reduce the noisy and irrelevant responses, we adapt the 2D GAU module to 3D GAU module in our decoder stage, which combines low-level features and high-level features for image recovery.

**Figure 4:**
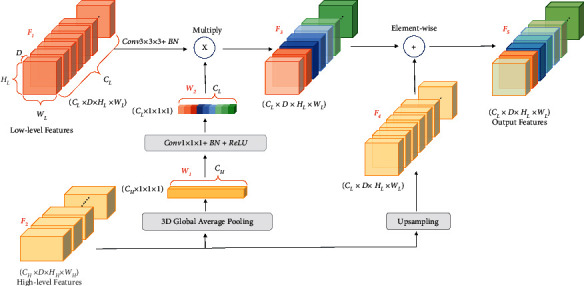
3D global attention upsample module.

The 3D GAU module is depicted in Figure 4. Low-level features are weighted by high-level features to select precise resolution details since the high-level features have abundant category information. On the high-level path, the 3D global average pooling operation extracts the category information contained in channels, generating the weights *W*_1_. Then the weights *W*_1_ are regarded as the attention weights from the high-level features, which will be multiplied with low-level features *F*_1_ after a convolution (kernel: [1, 1, 1], stride: [1, 1, 1], channels: *C*_*L*_) with batch normalization and ReLU nonlinearity operations, generating *F*_3_. The high-level feature map *F*_2_ is upsampled to *F*_4_. This upsampling process is achieved by a 3D transposed convolution (kernel: [1, 4, 4], stride: [1, 2, 2], channels: *C*_*L*_) and a batch normalization. Finally, the *F*_3_ and the *F*_4_ will be element-wisely added up, generating the final output *F*_5_.

### 3.4. Arrangement of Attention Modules

**Figure 5:**
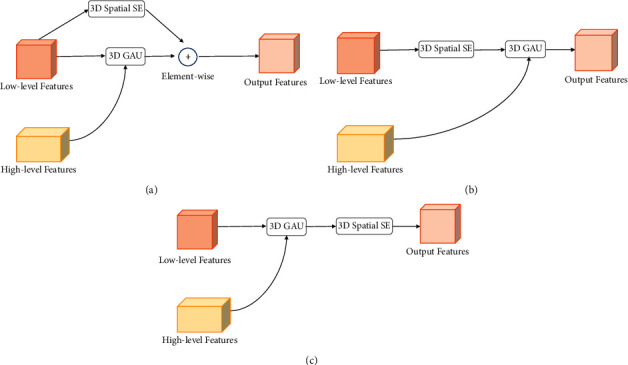
Three different arrangements of attention modules. (a) Spatial and channel attention in parallel manner. (b) Spatial first order of sequential manner. (c) Channel first order of sequential manner.

The role of channel attention mechanism and spatial attention mechanism is complementary. These two types of attention mechanisms are usually used in combination [41]. We initially performed an element-wise summation of the output of 3D spatial SE module and 3D GAU module (the architecture is depicted in Figure 5(a)). In this way, the two modules are placed in a parallel manner, but the experimental results were less effective. Inspired by CBAM [42], we tried the method of sequentially cascading two attention modules (Figures 5(b) and 5(c)). It was found that the sequential manner arrangement gives a better result than a parallel manner arrangement. And the experimental results indicate that the spatial first order is slightly better than the channel first order. In low-level features, 3D spatial SE module improves the feature expression ability of the network, and the output features are further guided and modified by high-level features channelwise through 3D GAU modules. In this way, the detailed information such as the boundary of the semicircular canal can be captured more accurately. The detailed experimental results of the different arrangement of attention modules are shown in Section 5.2.

### 3.5. Loss Function

In the training stage, we use the Dice similarity coefficient (DSC) loss function to constraint the network. The DSC loss function is shown in

(3) $$\begin{array}{c} L\left(G,P\right)=1-\frac{2\times \sum_{i}^{n}p_{i}g_{i}}{\sum_{i}^{n}p_{i}+\sum_{i}^{n}g_{i}}, \end{array}$$

where *P* and *G* represent predicted value and the ground truth, respectively, and *n* is the number of voxels; *p*_*i*_ and *q*_*i*_ denote the number of voxels in the *i*_th_ voxel in predicted data and ground truth, respectively.

### 3.6. Implemental Details

Our proposed network is implemented using the Pytorch package. Due to its efficiency, all the training and experiments were run on a standard workstation equipped with 16 GB of memory, an Intel(R) Core(TM) i5-10400F CPU working at 2.9 GHz, and a single NVIDIA RTX2060S GPU with 8 GB memory. The size of the raw CT image is 512 × 512 × 64. Due to the limitation of the GPU memory, we have to crop the volume into small patches as input to the network. To ensure a sufficient number of positive samples, we randomly crop the volume into 64 × 64 × 32 while ensuring that the cropped volume contains a part of semicircular canal. The batch size is set to 8; optimizer Adam is employed to speed training convergence. To reduce overfitting, we apply a weight decay of 0.0005 and a momentum of 0.97. The learning rate is set to 1 × e^−4^, and dice loss is the loss function. The training process is monitored by the validation accuracy.

### 3.7. Inference

**Figure 6:**
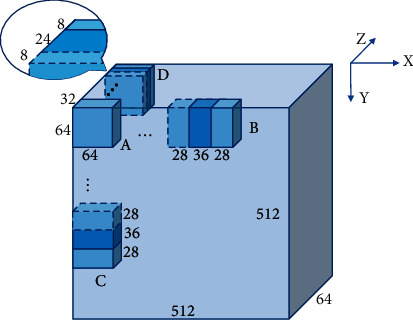
The inference method. The feature block with a size of 512 × 512 × 64 represents the original image; the smaller block A with the size of 64 × 64 × 32 represents the feature block that is input into the network for evaluation. B, C, and D, respectively, represent the sliding mode in the X-, Y-, and Z-axis directions, and the sliding steps are 28, 28, and 8, respectively.

Due to the memory limitations, we crop patches of size 64 × 64 × 32 as input to the network. To evaluate the performance of the model, each volume of the validation set or test set is sequentially cropped and evaluated to predict the entire image. As shown in Figure 6, we use 28 pixels as the step size for sliding in the *X*- and *Y*-axis while the step size is set to 8 in *Z*-axis. The volumes are cropped and evaluated after each slide. Due to sliding, there are overlapping areas where voxels will be evaluated repeatedly, and the repeated evaluation results of a specific voxel will be added up. We use a matrix to record the number of times each voxel is evaluated during the sliding process. The final evaluation result is divided by this matrix to get an average evaluation result. After the sigmoid function is processed, the threshold method is used to obtain the binarized prediction results.

## 4. Experimental Results and Dataset

### 4.1. Dataset

**Figure 7:**
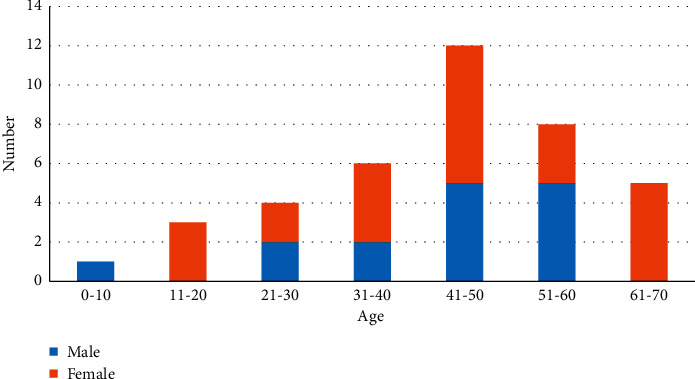
The age and gender distribution of the dataset samples. The abscissa numbers represent the age interval; the numbers on the ordinate indicate the corresponding number of people. Females are marked in orange and males are marked in blue.

**Figure 8:**
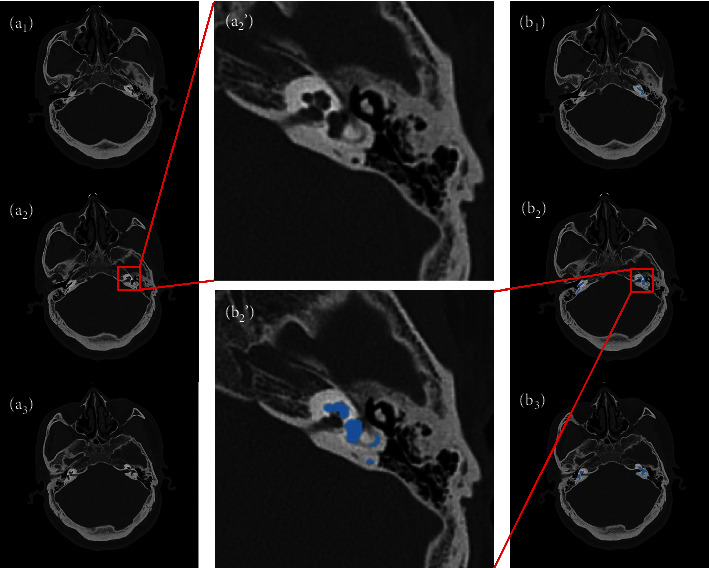
CT slices of our dataset. (a_1_), (a_2_), and (a_3_) represent the 37th, 39th, and 41st slices of a sample in our dataset. (b_1_), (b_2_), and (b_3_) are the corresponding labels; the areas where the semicircular canals are located are marked in blue. (a_2_') and (b_2_') are enlarged pictures of (a_2_) and (b_2_).

We collected 39 cases of raw semicircular canal CT images, which are voxel-level manually annotated by clinically experienced doctors. The age and gender distribution of the dataset samples are shown in Figure 7. The private information of the patients is encrypted. The size of each raw semicircular canal CT image is 512 × 512 × 64 voxels, the spacing of CT volumes in X, Y, and Z direction is 1 mm. The manual annotated CT images are used to train and objectively test our segmentation network. In 39 annotated cases, 26 cases are used as the training set and 7 cases are used as the validation set, and the other 6 cases are used as the test set. Some CT slices are shown in Figure 8. The three pictures in the left column, respectively, represent the 37th, 39th, and 41th original slices, while the three pictures in the right column are the corresponding ground truth (the regions of semicircular canals have been marked in blue). The two enlarged images in the middle represent the enlargement of the original image and the corresponding ground truth of the 39th slice.

### 4.2. Evaluation Metrics

We employed Dice similarity coefficient (DSC [%]), Average Hausdorff Distance (AVD [mm]), and Average Symmetric Surface Distance (ASD [mm]) to evaluate our proposed method in this paper. *P* and *G*, respectively, represent predicted value and the ground truth in the following descriptions.

DSC value indicates the overlapped voxels between the predicted results and the ground truth. And its mathematical definition is as shown in

(4) $$\begin{array}{c} \text{DSC}\left(P,G\right)=\frac{2\left|P\cap G\right|}{\left|P\right|+\left|G\right|}, \end{array}$$

where |·| denotes the number of labeled voxels. The larger the value of DSC, the higher the degree of overlap between the segmentation prediction and the ground truth.

Hausdorff Distance (HD) is also an evaluation index and it is frequently used in image segmentation. The Average Hausdorff Distance (AVD) is the Hausdorff Distance averaged over all points, which is more stable and less sensitive than HD. Its definition can be formulated as

(5) $$\begin{array}{c} \text{AVD}\left(P,G\right)=\max\left(d\left(P,G\right),d\left(G,P\right)\right), \end{array}$$

where *d*(*P*, *G*) is directed Average Hausdorff Distance that is given by

(6) $$\begin{array}{c} d\left(P,G\right)=\frac{1}{N}\sum_{p\in P}\underset{g\in G}{\min}\left\|p-g\right\|, \end{array}$$

where *N* is the number of voxels in *P*.

ASD is a surface-based metric to measure the average surface distance between symmetrical positions of two three-dimensional objects, which is *R*, and the Euclidean distance to the closest surface voxel from the ground truth *G* is calculated, and vice versa. Let *S* (*R*) denote the set of surface voxels of *R*; the shortest distance of an arbitrary voxel *v* to *S* (*R*) is defined as

(7) $$\begin{array}{c} d\left(v,S\left(R\right)\right)=\min_{s_{R}\in S\left(R\right)}\left\|v-s_{R}\right\|, \end{array}$$

where ‖·‖ denotes the Euclidean distance. Based on this, the ASD could be defined as follows:

(8) $$\begin{array}{c} \text{ASD}\left(R,G\right)=\frac{1}{\left|S\left(R\right)\right|+\left|S\left(G\right)\right|}\times \left(\underset{S_{R}\in S\left(R\right)}{\sum }d\left(S_{R},S\left(G\right)\right)+\underset{S_{G}\in S\left(G\right)}{\sum }d\left(S_{G},S\left(R\right)\right)\right), \end{array}$$

for ASD values, the smaller the better.

### 4.3. Experimental Results

We evaluated the model on the test dataset which contains 6 samples, and the results are shown in Table 1. It can be seen that our network has a good performance on the test dataset. Most samples reach a dice coefficient over 90%. However, the segmentation result of sample 4 is not ideal. As a matter of fact, in sample 4, the superior semicircular canal part of the left semicircular canal is less segmented in the prediction. The specific analysis is discussed in Section 5.4.

**Figure 9:**
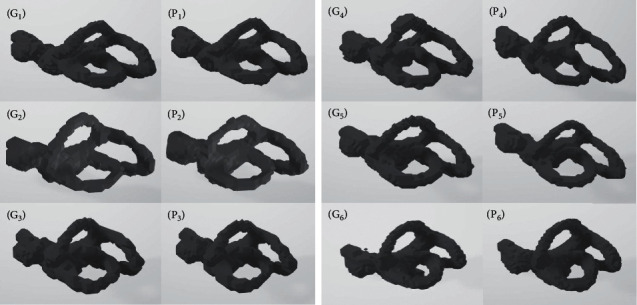
The segmentation results of the test dataset. *G*_1_, *G*_2_,…, *G*_6_ represent the ground truth of the 6 samples in the test set, while *P*_1_, *P*_2_,…, *P*_6_ are the corresponding predictions. All of the semicircular canals are the right semicircular canal.

The visualization results are shown in Figure 9. The ground truth image is on the left, while the predicted image is on the right. The ground truth and predictions corresponding to the same semicircular canal are, respectively, marked with *G*_*i*_ and *P*_*i*_(*i*=1,2,…, 6). For the convenience of presentation, we only show one of each pair of semicircular canals.

## 5. Discussion

Ablation experiments are conducted to evaluate the effectiveness of the 3D spatial SE module and the 3D GAU module in the proposed network. We first add the 3D spatial SE module and 3D GAU module to 3D U-Net separately to verify the effectiveness of them and then use them at the same time to verify that the combination of the two can make the network perform better. And in 5.2, we compare three different ways of arranging the channel and spatial attention submodules and discuss why we choose the spatial first-order sequential arrangement. For further comparison, we train models using other state-of-the-art deep learning architectures with the same training settings on our dataset. The experimental results show that our method achieves better performance.

### 5.1. Ablation Experiments

The results of the ablation experiments are shown in Table 2. The well-known 3D U-Net is used as the benchmark. We replace the decoder stage of 3D U-Net with 3D GAU modules, and we call it 3D GAU U-Net. We introduced 3D spatial SE modules to each layer of the encoder and named it 3D sSE U-Net. Finally, we simultaneously introduce the 3D GAU modules and the 3D spatial SE modules in 3D U-Net as our network. Experimental results show that our proposed network has the best performance.

Comparing the segmentation performance of the 3D U-Net with 3D GAU U-Net, we find that when the 3D GAU modules are introduced, the DSC value increases from 91.6% to 91.9%, the AVD value drops by 0.301 mm, and the ASD value drops by 1.30 mm. This result proves that the 3D GAU module is able to improve the segmentation accuracy.

To capture the spatial dependencies among positions of the feature maps from the encoder, we add the 3D spatial SE attention module in the network and this operation is proved to be beneficial for our segmentation task. According to Table 2, the DSC value of 3D sSE U-Net increases by about 0.6% to the 3D U-Net, and the AVD and ASD values decrease by 0.822 mm and 3.44 mm, respectively. The results above indicate that the 3D spatial SE attention module helps to enlarge the receptive field and brings benefits to the segmentation results.

We combine the 3D GAU module with the 3D spatial SE attention module and find out that this combination can achieve the best result: the DSC value is increased to 92.5%; the AVD and ASD achieve 0.217 mm and 0.639 mm, respectively. This experiment proves the effectiveness of our improvements.

**Figure 10:**
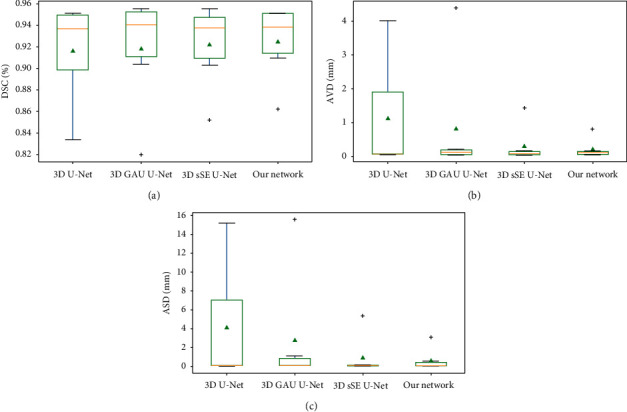
Ablation experiment statistics box diagram of semicircular canals on the test dataset. (a) The DSC results of four methods. (b) The AVD results of four methods. (c) The ASD results of four methods. The compartments between the boxes represent the upper quartile and the lower quartile data distribution intervals. The green triangle, black plus sign, and the orange line represent the mean, outlier, and median, respectively.

We use the box chart to calculate the distribution of the segmentation results of 6-test data with different methods.  Figure 10 indicates that our method achieves relatively higher mean DSC value, and the variation is within the acceptable range. The AVD and ASD values of our method results are lower with small variations. The 3D sSE U-Net with 3D spatial SE module has the competitive performance to our network, but our network combines the advantages of spatial and channel attention mechanism, which not only improves the average segmentation accuracy, but also reduces the variance, making the model more robust.

### 5.2. Combining Methods of Channel and Spatial Attention Modules

In this experiment, we compared three different ways of arranging spatial attention modules and channel attention modules: channel first-order sequential arrangement, spatial first-order sequential arrangement, and parallel use of both attention modules.  Table 3 summarizes the experimental results on different attention arranging methods.

As shown in Table 3, arranging the attention modules in sequential manner achieved better performance than in parallel manner. Theoretically, there is a huge difference between the outputs of the channel attention module and the spatial attention module; thus in parallel manner arrangement mode the simple direct summation of the two output features will mix different information and adversely affect the training process. According to the experiment results, the spatial first order is slightly better than channel first order, and we adopt the better one as our method.

### 5.3. Comparison with Other Networks

In this section, the proposed network architecture is compared with several state-of-the-art deep-learning-based architectures. We perform V-Net [21], Residual Symmetric U-Net [22], DenseVoxNet [23], and VoxResNet [19] on our dataset with the same training settings as our method. The experimental results are shown in Table 4. The dice coefficient of V-Net is 86.3%, which is the lowest among the networks. Residual Symmetric U-Net reaches the dice score of 91.7% which is close to our result, but the AVD and ASD results are worse. DenseVoxNet and VoxResNet, respectively, achieved 88.3% and 89.4% dice coefficient, and both networks perform well on big organs, while they are less effective on the small organ task. It shows that our network achieves state-of-the-art performance in the task.

### 5.4. Limitations and Future Work

**Figure 11:**
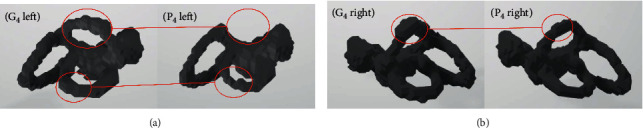
The comparison of ground truth and prediction of sample 4. (a) The left semicircular canal of sample 4, where (*G*_4_ left) is the ground truth and (*P*_4_ left) is the corresponding prediction. (b) The right semicircular canal of sample 4, where (*G*_4_ right) is the ground truth and (*P*_4_ right) is the corresponding prediction. The difference between the ground truth and the prediction is marked with the red circle.

Although our network can achieve good segmentation results on most samples, the dice coefficient of sample 4 in Table 1 is less than 90%, which is far from satisfactory.  Figure 11(a) is the left and Figure 11(b) is the right semicircular canal of sample 4. It shows in Figure 11(a) that the superior semicircular canal part of the left semicircular canal is completely missing. In other parts, there are also some differences between ground truth and predictions. There are several reasons that may account for this. Our network was trained on a training cohort with an age range from 17 to 70. However, in the test dataset, sample 4 is from a nine-year-old child, whose semicircular canal is about 30% smaller than other adult samples; thus it is difficult to segment sample 4 with the same CNN settings. The diameter of the superior semicircular canal is less than 8 pixels in the image. After three times of pooling, that is, 8 times of downsampling, its information merges with the background. Due to the loss of this information, the network cannot restore the shape of the superior semicircular canal during the decoder stage. Another possible reason is that the semicircular canals of children are not fully developed, so their morphological differences from adults may lead to the inaccurate segmentation in some details. Nevertheless, our network has better segmentation performance on sample 4 than other state-of-the-art networks, the results of sample 4 are listed in Table 5; it shows that the segmentation dice coefficient of our network for sample 4 is at least 2.8% higher than other networks.

In the future, we will enlarge our training set by collecting data from different age groups. Meanwhile, we will design an extra branch to adapt the original network to smaller size samples, which would improve the robustness and generalization of the network. And our proposed network will be applied in other segmentation tasks in the future.

## 6. Conclusions

This paper proposes an improved 3D U-Net with attention mechanism, which can segment semicircular canal in CT volumes effectively. The network makes extensive use of the volumetric data information, improves the feature utilization, and reduces the information loss by replacing the skip connections of 3D U-Net with spatial and channel attention modules in a spatial first-order manner. The performance of the proposed CNN network achieved a mean DSC of 92.5%, a mean AVD of 0.217 mm, and a mean ASD of 0.639 mm on the test set. The experimental results indicate that the proposed method achieves better results than some state-of-the-art methods. The accurate segmentation of semicircular canal helps to promote the artificial intelligence-assisted diagnosis of ear diseases, inner ear surgical planning, and customized reduction treatment of otolithiasis.

## Figures and Tables

**Table 1:** The evaluation results of the network.

Samples	DSC (%)	AVD	ASD
Sample 1	95.05	0.0528	0.0443
Sample 2	92.64	0.0999	0.0589
Sample 3	95.11	0.0595	0.0585
Sample 4	86.21	0.8142	3.0773
Sample 5	95.03	0.1266	0.5376
Sample 6	90.97	0.1515	0.0625
Mean	92.50	0.2174	0.6399

**Table 2:** The results of the ablation experiments.

Scenarios	DSC (%)	AVD	ASD
3D U-Net	91.6	1.130	4.130
3D GAU U-Net	91.9	0.829	2.830
3D sSE U-Net	92.2	0.308	0.944
Our network	92.5	0.217	0.639

**Table 3:** The results of different arrangements of attention modules.

Description	DSC (%)	AVD	ASD
3D U-Net + channel + spatial	91.9	0.444	1.515
3D U-Net + spatial + channel	92.5	0.217	0.639
3D U-Net + channel and spatial in parallel	91.3	1.145	4.675

**Table 4:** The results of comparison with other networks.

Network	DSC (%)	AVD	ASD
V-Net	86.3	5.014	12.00
Residual Symmetric U-Net	91.7	1.828	5.439
DenseVoxNet	88.3	0.595	2.345
VoxResNet	89.4	1.626	5.956
Our network	92.5	0.217	0.639

**Table 5:** The results of sample 4 of different networks.

Network	DSC (%)	AVD	ASD
V-Net	65.4	25.94	57.154
Residual Symmetric U-Net	82.7	4.839	11.059
DenseVoxNet	81.6	2.233	9.906
VoxResNet	82.5	3.774	15.673
3D U-Net	83.4	4.007	15.187
Our network	86.2	0.814	3.077

## Data Availability

The training data and test data used to support the findings of this study were supplied by the First Affiliated Hospital of Guangzhou Medical University under license and so cannot be made freely available. Requests for access to these data should be made to Prof. Xiaowen Zhang, entxiaowen@vip.163.com.
